# *Limosilactobacillus reuteri* ATG-F4 Improves the Muscle Strength and Muscle Mass of Mice with Immobilization-Induced Muscular Atrophy

**DOI:** 10.4014/jmb.2506.06004

**Published:** 2025-10-15

**Authors:** Daeyoung Lee, Young-Sil Lee, Gun-Seok Park, Juyi Park, Seung-Hyun Ko, You-Kyung Lee, Do Yeun Jeong, Yong Hyun Lee, Jihee Kang

**Affiliations:** Atogen Co., Ltd., Daejeon, Republic of Korea

**Keywords:** *Limosilactobacillus*, probiotics, muscle atrophy, inflammation, gut microbiota, short-chain fatty acids

## Abstract

Probiotics offer a promising avenue for combating muscle atrophy, which is a debilitating condition associated with muscle disuse, aging, and disease. This study investigated the anti-atrophic potential of *Limosilactobacillus reuteri* ATG-F4, a human gut-derived bacterium, in a mouse model of staple-induced immobilization. ATG-F4 administration significantly preserved muscle mass and improved grip strength and endurance. Mechanistically, ATG-F4 activated mTOR signaling and promoted protein synthesis. Additionally, ATG-F4 downregulated MuRF1, a key atrophy factor. Furthermore, ATG-F4 administration significantly altered the composition of the gut microbiota, favoring the presence of the Muribaculaceae family and decreasing the abundance of Lachnospiraceae and Lactobacillaceae. Administration of ATG-F4 increased serum levels of the short-chain fatty acids (SCFAs) butyrate and acetate. SCFAs, which are produced by bacterial fermentation in the gut, possess anti-inflammatory and beneficial muscle properties and exert several effects on host metabolism and the immune system. Therefore, we suggest that the potential mechanism underlying the anti-atrophic effects of ATG-F4 on muscles involves the enhancement of muscle protein synthesis, suppression of protein degradation, and modulation of the gut-muscle axis. These findings highlight the potential of ATG-F4 as a prophylactic or therapeutic agent for the treatment of muscle atrophy.

## Introduction

The skeletal muscle is an important tissue that moves the human body and performs a variety of functions, such as storing carbohydrates as an energy source and maintaining body temperature. However, muscle mass can easily be lost depending on nutritional status, hormonal balance, physical activity, injury, illness, and aging. Muscle atrophy occurs in patients with diseases such as cancer, acquired immunodeficiency syndrome (AIDS), Duchenne muscular dystrophy (DMD), renal and heart failure, chronic obstructive pulmonary disease (COPD), and diabetes [1-5]. Muscle atrophy negatively affects muscle function and longevity, and deteriorates the quality of life because muscle mass influences general metabolism, locomotion, and respiration [6]. Therefore, research aimed at the prevention and treatment of muscle atrophy is critical for human health.

Emerging evidence has demonstrated a relationship between the gut microbiota and skeletal muscles [7, 8]. Studies have explored the "gut-muscle axis" to support the hypothesis that gut microbiota imbalance contributes to the onset and progression of age-related muscle atrophy by promoting systemic inflammation [9-11]. Building on this, other studies specifically examined the role of the gut microbiome in regulating skeletal muscle mass and physical function by highlighting short-chain fatty acids (SCFAs) and the influence of specific beneficial bacteria such as *Lacticaseibacillus casei* as key signaling pathways [12]. Furthermore, one study provided experimental evidence in obese and type 2 diabetic mouse models, showing that prebiotic-induced changes in the gut microbiota not only improved glucose and lipid metabolism, but also successfully increased muscle mass [13].

Probiotics represent one of the fastest-growing categories in functional health supplements, with scientifically validated therapeutic benefits [14, 15]. Some probiotic strains promote gut microbiome health, which can help prevent common diseases such as obesity, diabetes, autism, osteoporosis, and certain immune disorders [16]. Probiotics can affect various organs such as the brain, liver, lungs, bones, and muscles [17-21]. Probiotics are unable to act directly on organs outside the digestive system; therefore, it is assumed that changes in metabolites or immune markers, which result from the modulation of the gut microbiota by probiotics, may affect other organs.

In this context, targeting the gut-muscle axis with probiotics is one strategy to prevent or improve the health of the host muscle. For instance, *Bifidobacterium breve* B-3 tends to increase muscle mass by activating the mTOR-p70S6K signaling pathway in muscles [22]. *Akkermansia muciniphila* and *Faecalibacterium prausnitzii* treatments decrease the expression levels of the ubiquitin-proteasome genes atrogin-1, MuRF1, and cathepsin L and increase the expression of mitochondrial biogenesis regulatory genes [23]. Collectively, these reports suggest that probiotics are candidate therapeutic agents against muscle weakness and impairment.

*Limosilactobacillus reuteri* is a probiotic species that contributes to maintaining the intestinal microbial balance and exerts systemic health-promoting effects across multiple organs and systems [24, 25]. Recent preclinical and basic studies have suggested that *L. reuteri* may exert beneficial effects on muscle atrophy [26-28]. However, the underlying biological mechanisms and clinical applications have not been widely studied. The *L. reuteri* ATG-F4 strain used in this study was isolated from newborn feces and was suggested as a psychobiotic candidate by demonstrating anti-inflammatory effects, serum dopamine modulation, gut microbiota, and gut circadian rhythm modulation in a healthy mouse model [29]. Based on these results, we assumed that ATG-F4 could have a positive effect on muscle atrophy, because several studies have suggested that the putative neural mechanism is related to muscle weakness and that dopamine in the central nervous system is an essential regulator of mobility, performance, and vigor [30-32].

In this study, we investigated the potential of *L. reuteri* ATG-F4 for treating muscle atrophy.

## Materials and Methods

### Preparation of *L. reuteri* ATG-F4

*L. reuteri* ATG-F4 (ATG-F4) was isolated from the feces of a newborn [29]. ATG-F4 was cultured in de Man Rogosa Sharpe (MRS) medium (Difco Laboratories, USA) at 37°C for 24 h. ATG-F4 cultures were centrifuged at 4,000 ×*g* for 10 min to obtain the bacterial cells that were used in animal experiments.

### Bioethics Declaration

This animal study was approved by the Institutional Animal Care and Use Committee (IACUC) of AtoGen Co., Ltd. (approval no. ATG-IACUC-RDSP-200713).

### Animals and Experimental Groups

Five-week-old C57BL/6J male mice were purchased from Central Laboratory Animal Inc. Republic of Korea). The facility was maintained at 23 ± 2°C and a humidity of 55 ± 10%, with a 12 h/12 h light/dark cycle. The mice were fed a normal diet (Purina. cat no. 38057, Cargill, Republic of Korea) and provided with sterilized distilled water. After a one-week acclimation period, the mice were randomly divided into three treatment groups: untreated (*n* = 10), stapled (*n* = 10), and stapled + ATG-F4 (*n* = 10).

ATG-F4 was administered orally at approximately 4.0 × 10^9^ CFU/mouse for 27 days, whereas the untreated and stapled groups were administered Phosphate-buffered saline. On the 14th day, the right leg of each mouse in both the stapled and stapled + ATG-F4 groups was fixed for 10 days using a staple. Three days after the staple removal, fresh fecal samples were collected from each mouse in an empty cage. Additionally, wire-hanging and treadmill tests were performed on all animals to assess improvements in muscle rehabilitation and exercise capacity owing to ATG-F4 consumption.

All animals were sacrificed the day after the muscular performance test. Blood samples were collected after euthanasia by CO_2_ inhalation, and freshly obtained serum samples were processed for cytokine and SCFAs analyses. Five types of leg skeletal muscles — tibialis anterior (TA), gastrocnemius (GA), plantaris (PL), extensor digitorum longus (EDL), and soleus (SOL) — were separated and weighed. All muscle tissues were stored at -70°C until analyzed.

### Wire Hang Test

As described by Deacon [33], the wire hang test was performed using a 440 mm × 330 mm wire mesh composed of 10 mm^2^ squares. Time was measured from the moment the wire screen, with the mouse placed at the center, was turned over. The screen was fixed at least 40 cm above the padded surface to absorb shock from falling mice. All animals were warmed for 3 min on a wire without any weight. During the wire hang test, each mouse was allowed to hang for up to 300 s with a weight equivalent to approximately 25% of its body weight attached, and hang time was recorded. The wire hang test was repeated three times independently. The falling times of the mice were compared and analyzed using the average values. Furthermore, the falling time was reevaluated as follows: falling time between 1 and 60 s = 1, between 61 and 120 s = 2, between 121 and 180 s = 3, between 181 and 240 s = 4, between 241 and 300 s = 5, and after 300 s = 6.

### Treadmill Test

The treadmill test was conducted using a 5-lane treadmill (Harvard Apparatus, USA). Before the experiment, the mice were warmed to 8 m/min for 5 min. Electrical stimulation was initially set to 1.25 mA to trigger running. For the actual test, the speed was set to 10 m/min and automatically increased by 2 m/min every 10 min. The running was performed for up to 80 min in eight steps. After 30 min, the inclination angle of the treadmill was increased by 2°C every 10 min, reaching 10°C. After the test was completed, both running time and distance were recorded for analysis.

### Cytokine Assay

The levels of TNF-α and IL-6 in the serum and the supernatant of crushed muscle tissues were measured using a mouse TNF-α and IL-6 ELISA max standard set (BioLegend, USA). The cytokine levels in TA and GA muscles were normalized to the total protein concentration in the samples (determined using Bicinchoninic Acid assays) and expressed as pg/mg.

### Western Blotting Analysis

Flash-frozen TA and GA muscles were thoroughly crushed in liquid nitrogen. RIPA buffer (0.5 M Tris-HCl, pH 7.4, 1.5 M NaCl, 2.5% deoxycholic acid, and 10% NP-40) containing a protease inhibitor cocktail (Millipore, USA) was added to the crushed muscle tissues after the liquid nitrogen evaporated. The homogenate was centrifuged at 14,000 ×*g* for 10 min at 4°C, and the supernatant was recovered. Protein concentrations in the supernatants were determined using BCA assays (Thermo Fisher Scientific, USA). Protein extracts (40 μg-containing aliquots) were separated on 8 or 10% polyacrylamide mini gels and transferred to PVDF membranes (Bio-Rad, USA). Blotted membranes were incubated overnight at 4°C in SuperBlock (PBS) blocking buffer containing Kathon antimicrobial agent. The membranes were then probed with primary antibodies against mTOR, p-mTOR, p70S6K, p-p70S6K, rpS6, p-rpS6, Atrogin-1, MuRF1, and β-actin (1:1000 dilution; Cell Signaling Technology, USA). The blots were washed four times with 0.1% Tween TBS and incubated for 1 h at approximately 22–25°C with goat anti-rabbit IgG HRP-conjugated secondary antibody (Bio-Rad) in 0.1% Tween TBS buffer containing 3% BSA (Bovogen Biologicals, Keilor East, Australia). After extensive washing with 0.1% Tween TBS, the immunostained bands were revealed with ECL (Bio-Rad). The target complex was detected using a ChemiDoc Imaging System (Bio-Rad). Target band intensity was quantified using Image Lab software (Bio-Rad).

### Real-Time PCR

Total RNA was extracted from flash-frozen TA and GA muscles using the TRIzol reagent (Thermo Fisher Scientific) after crushing in liquid nitrogen. The homogenate was mixed with chloroform and centrifuged at 13,000 ×*g* for 5 min at 4°C. The supernatant was then collected, and isopropanol was added to a final concentration of 70%. After centrifugation at 13,000 ×*g* for 5 min at 4°C, the pellet was washed twice with 70% ethanol (instead of isopropanol) to obtain the RNA precipitate. DEPC water was added to the RNA precipitate. cDNA was synthesized from 1 μg of RNA using ccPower RT PreMix (Bioneer, Republic of Korea). For PCR amplification, the following primers were used: for Atrogin-1, 5'-ACACTGGTGCAGAGAGTCGG-3' (forward) and 5'-TAAGCACACAGG CAGGTCGG-3' (reverse); for MuRF1: 5'-GCGTGACCACAGAGGGTAAAGA-3' (forward) and 5'-GTGGGG AGCCCTATGCTAGTC-3' (reverse); and for β-actin: 5'-TCTACGAGGGCTATGCTCTCC-3' (forward) and 5'-GGATGCCACAGGATTCCATAC-3' (reverse). Quantitative RT-PCR was performed by mixing 10 ng of cDNA with PowerUP SYBR Green Master Mix (Thermo Fisher Scientific) using a 7500 Fast Real-Time PCR System (Thermo Fisher Scientific). qPCR conditions included an initial 20 sec denaturation step at 95°C, followed by 45 cycles at 95°C for 3 sec and 60°C for 30 sec.

### Histology

TA and GA muscles were excised and fixed in 10% formalin for more than 48 h at 20-25°C. The fixed tissues were subjected to general tissue processing procedures such as trimming, dehydration, paraffin embedding, and thinning to prepare them for histopathological examination, and then stained with hematoxylin and eosin (H&E). Histopathological changes were observed using 400× enlarged H&E slide microphotographs captured using a camera attached to an optical microscope (Olympus BX53, Japan). Myofiber size was obtained from the cross-sectional area (CSA) of the H&E-stained sections. More than 30 myofibers were examined per field from three perspectives.

### Fecal Microbiota Analysis

Genomic DNA was extracted from fecal samples using a QIAamp PowerFecal Pro DNA Kit (Qiagen, Germany). The quantity and quality of extracted DNA were measured using a Qubit 3.0 fluorometer (Thermo Fisher Scientific) and agarose gel electrophoresis, respectively. The V4 hypervariable regions of the bacterial 16S rRNA were amplified with unique 8-bp barcodes and sequenced on an Illumina MiSeq 100 system platform according to the standard protocol [34]. Raw reads were analyzed using the QIIME2 pipeline [35]. Sequences were quality filtered and clustered into operational taxonomic units with 97% sequence identity, according to the SILVA 132 database [36]. Operational taxonomic units were identified from the phylum to the family level.

### Short-Chain Fatty Acid Analysis

Analytical standards of SCFAs, including acetate (≥ 99.8% purity), butyrate (≥ 99.5% purity), and propionate (≥ 99.5% purity), were purchased from Sigma-Aldrich (USA). Ammonium formate (≥ 99.995% purity) and formic acid (≥ 99.5% purity), as well as high-performance liquid chromatography (HPLC)-grade methanol (MeOH), acetonitrile (ACN), and water, were also purchased from Sigma-Aldrich. A 100 μl aliquot of serum was mixed with 500 μl of ice-cold MeOH with 1% formic acid. The solution was mixed by vortex for 20 min and left for 2 h at 4°C to solidify the protein precipitate. After centrifugation at 14,000 ×*g* for 30 min at 4°C, the supernatant was filtered using a PVDF syringe filter (0.22 μm pore size) (Millipore, USA). The filtered sample was then injected into a liquid chromatography-tandem mass spectrometry (LC-MS/MS) system, including an Exion LC system connected to a QTRAP 4500 mass spectrometer (SCIEX, USA) and an Intrada organic acid analysis column (150 × 2 mm, 3-μm particle size, Imtakt, Japan). A 5 μl aliquot was used for the autosampler injection; the flow rate was set to 0.2 ml/min and the temperature to 40°C. Negative ion mode scanning of a gradient mobile phase consisting of (A) an acetonitrile/water/formic acid (10/90/0.1 *v/v*) solution and (B) an acetonitrile/100 mM ammonium formate (10/90 *v/v*) solution was used. The gradient started at 0% B and was maintained for 1 min; then, the gradient increased linearly over 6 min until 100% B was reached. The mobile phase composition was maintained at 100% B for 3 min before it was quickly returned to 0% B in 0.1 min. Finally, the gradient was maintained at 0% B for 4.9 min to re-equilibrate the column. The total analysis time was 15 min. The analysis was performed using an electrospray ionization source in the negative mode. The operating conditions were as follows: ion spray voltage, 4,500 V; curtain gas (CUR), 25 psi; collision gas (CAD), medium; ion source gas 1 (GS1) and ion source gas 2 (GS2), 50 and 50 psi, respectively; turbo spray temperature (TEM), 450°C; entrance potential (EP), 10 V; and collision cell exit potential (CXP), 5 V. Nitrogen was used in all cases. Analytes were quantified by multiple reaction monitoring (MRM) employing the following precursor to product ion transitions and parameters: acetate, m/z 105.0 → 44.9 with DP 5 V and CE 18 eV; butyrate, m/z 133.0 → 44.9 with DP 5 V and CE 18 eV; propionate, m/z 119.0 → 45.1 with DP 5 V and CE 16 eV. SCIEX OS 2.0.0 software was used for data acquisition and processing, and Analyst 3.3 software was used for data analysis.

### Statistical Analyses

One-way analysis of variance (ANOVA) with Dunnett’s test was performed for multiple comparisons using GraphPad Prism software (version 8.0). Differences were considered statistically significant at *p* < 0.05. For fecal microbiota analysis, the non-parametric Kruskal–Wallis test was used to compare differences in diversity indices and microbial taxa. All data are expressed as the mean ± SEM.

## Results

### ATG-F4 Preserves Skeletal Muscle Mass in Immobilized Mice

**Fig. 1:**
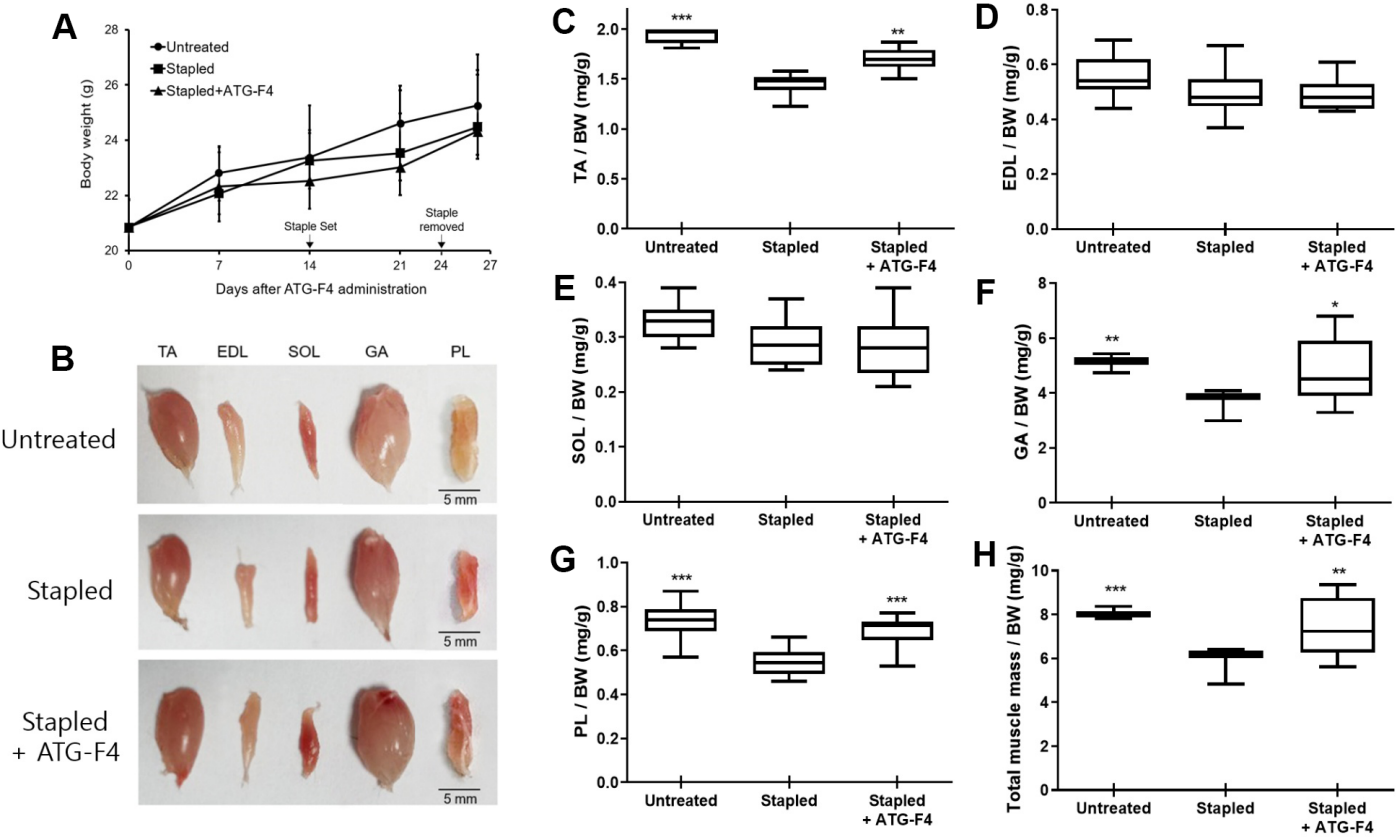
Body weight, hind limb muscle mass, and representative muscle images from mice from different groups. (**A**) Mice bodyweight; (**B**) mice hind limb images; and (**C–H**) relative weight of (C) TA, (D) EDL, (E) SOL, (F) GA, (G) PL, and (**H**) total mouse muscles. TA, tibialis anterior; EDL, extensor digitorum longus; SOL, soleus; GA, gastrocnemius; PL, plantaris; untreated, unstapled group; stapled, staple-induced immobilization group; stapled + ATG-F4, staple-induced immobilization + *L. reuteri* ATG-F4-treated group; BW, body weight. Data are presented as the mean ± SEM (*n* = 10 mice per group). *p < 0.05, **p < 0.01, and ***p < 0.001 compared to the stapled group.

The total body weights of the three mouse groups showed no significant differences during the experimental period (Fig. 1A). In contrast, the TA, GA, PL, EDL, and SOL muscles in the stapled group were atrophied by 28.9%, 30.3%, 28.0%, 20.7%, and 15.3%, respectively, compared to the corresponding muscle masses of mice in the untreated group. ATG-F4 administration resulted in a significant increase in muscle mass by 17.3%, 30.3%, and 27.0% in the TA, GA, and PL muscles, respectively, compared to that in mice from the stapled group (Fig. 1B-1G). Additionally, the total muscle mass of the hind limbs of mice in the stapled + ATG-F4 group was significantly augmented by 22.4% compared with that of mice in the stapled group (Fig. 1H).

### ATG-F4 Enlarges Muscle Fiber Size

**Fig. 2:**
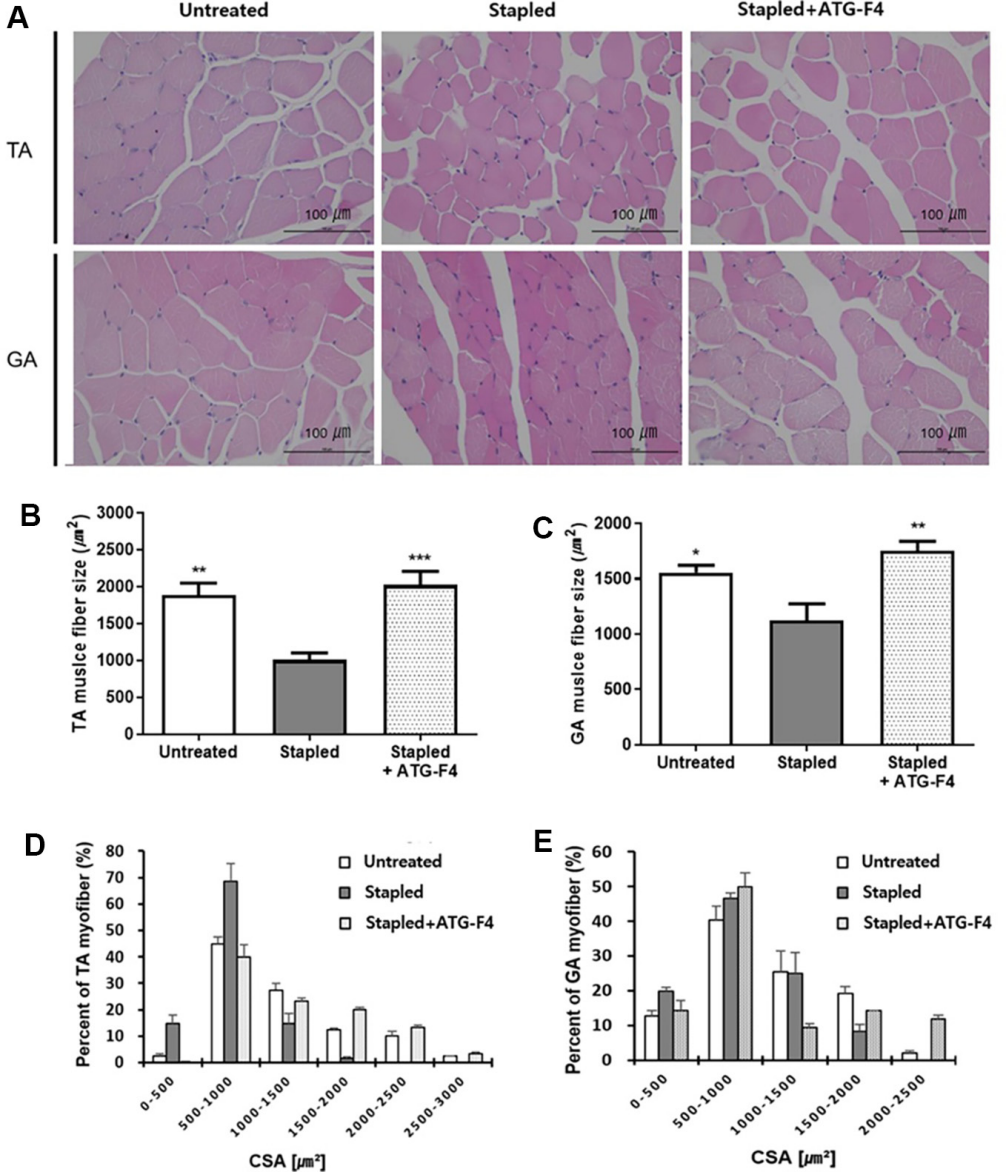
H&E staining of TA and GA muscle fibers of mice from different groups. (**A**) Representative images of TA and GA muscle fibers from mice of different groups; (**B–C**) CSA quantification in (**B**) TA and (**C**) GA muscle fibers; (**D–E**) myofiber size distribution in (**D**) TA and (**E**) GA muscles. TA, tibialis anterior; GA, gastrocnemius; untreated, unstapled group; stapled, staple-induced immobilization group; stapled + ATG-F4, staple-induced immobilization + *L. reuteri* ATG-F4-treated group; CSA, cross-section area. Data are presented as the mean ± SEM (*n* = 5 mice per group). *p < 0.05, **p < 0.01, and ***p < 0.001 compared to the stapled group.

The CSA of myofibers in the TA and GA muscles of each experimental group was analyzed using H&E staining (Fig. 2). The average size of myofibers in mice from the stapled group decreased compared to that in mice from the untreated group, whereas the size of myofibers in mice from the stapled + ATG-F4 group significantly increased compared to that in the stapled group (Fig. 2B and 2C). Moreover, there was a significant increase in the proportion of large myofibers in the TA and GA muscles of the stapled + ATG-F4 group compared with that in the TA and GA muscles of the stapled group (Fig. 2D and 2E).

### ATG-F4 Improves Muscle Strength and Endurance

**Fig. 3:**
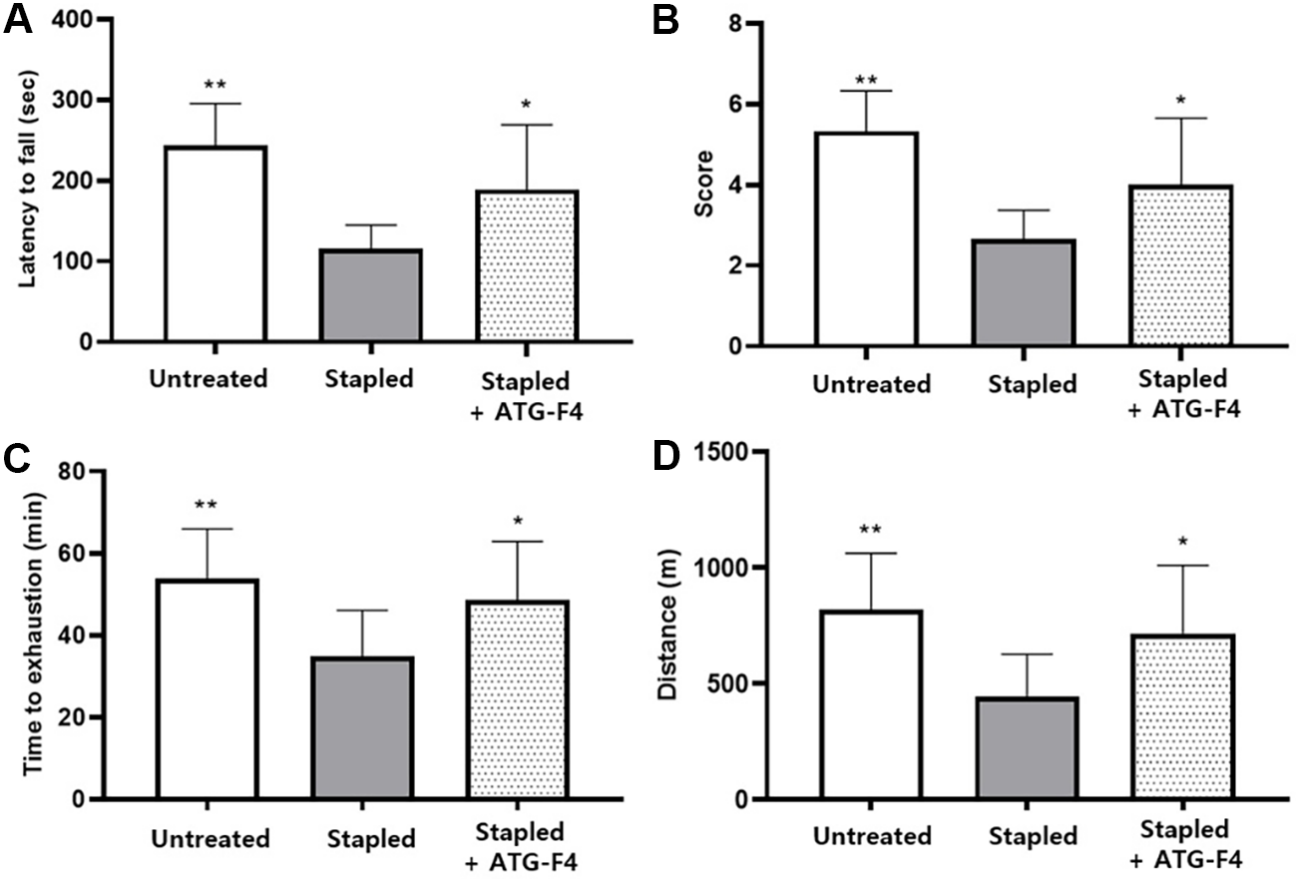
Physical performance tests measuring muscle strength and endurance in mice from different groups. (**A**) Latency to fall and (**B**) its corresponding scores from the wire hang test done for muscle strength determination in mice; (**C**) running time to exhaustion and (**D**) the corresponding distances in the treadmill test performed in mice from different groups. untreated, unstapled group; stapled, staple-induced immobilization group; stapled + ATG-F4, staple-induced immobilization + *L. reuteri* ATG-F4-treated group. Data are presented as the mean ± SEM (*n* = 10 mice per group). *p < 0.05 and **p < 0.01 compared to the stapled group.

To estimate muscle strength, we performed wire hang tests 3 days after staple removal. The latency to fall and average maximum score in ATG-F4 mice were significantly higher than in those of the stapled group, at 188.9 ± 80.2 vs. 116.1 ± 29.2 and 4.0 ± 1.7 vs. 2.7 ± 0.7, respectively (Fig. 3A and 3B). In the treadmill test assessment of functional muscle recovery, the time to exhaustion was significantly shorter in the stapled group than in the untreated group. In contrast, mice from the stapled + ATG-F4 group showed a significant increase in both running time and distance compared to those of mice from the stapled group, at 48.7 ± 14.2 vs. 34.9 ± 11.2 and 716.6 ± 294.5 vs. 445.9 ± 182.1, respectively (Fig. 3C and 3D).

### ATG-F4 Reduces Systemic and Local Inflammatory Cytokines

**Fig. 4:**
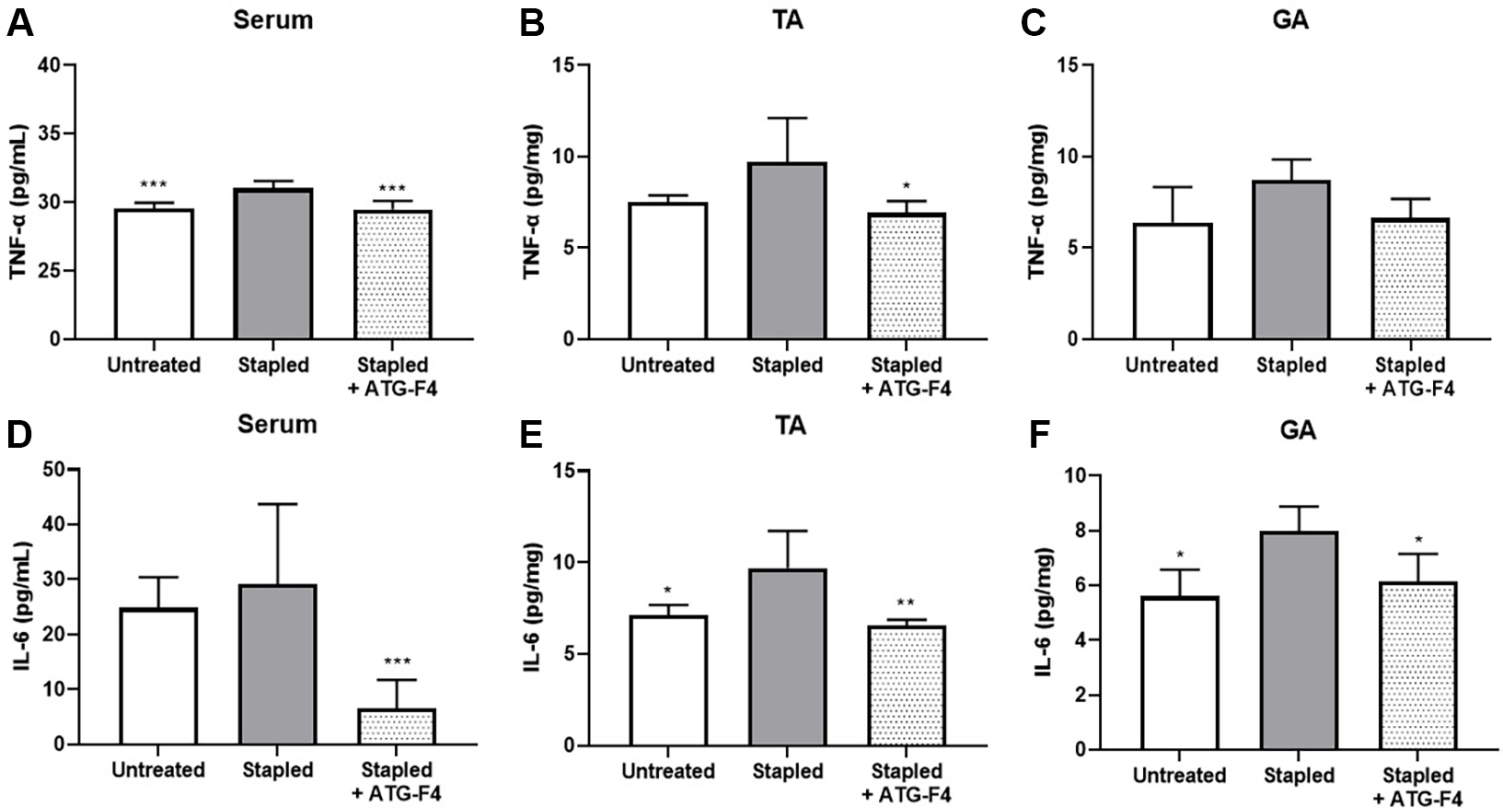
Levels of inflammatory cytokines in serum and muscle tissues of mice from different treatment groups. (**A–C**) Levels of TNF-α in (**A**) serum and (**B**) TA and (**C**) GA muscles of mice; (**D–F**) levels of IL-6 in (**D**) serum and (**E**) TA and (**F**) GA muscles of mice. TA, tibialis anterior; GA, gastrocnemius; untreated, unstapled group; stapled, stapleinduced immobilization group; stapled + ATG-F4, staple-induced immobilization + *L. reuteri* ATG-F4-treated group. Data are presented as the mean ± SEM (*n* = 10 mice per group). *p < 0.05, **p < 0.01, and ***p < 0.001 compared to the stapled group.

Inflammatory cytokine levels were measured in the serum and the TA and GA muscles of mice in all groups. TNF-α and IL-6 levels were higher in the serum and muscle tissues of mice from the stapled group than in those of mice from the untreated group. ATG-F4 administration significantly reduced TNF-α levels in the serum and TA muscle of mice compared to those in mice from the stapled group (Fig. 4A and 4B). However, no significant differences in TNF-α levels were observed in the GA muscles of mice from the different groups after ATG-F4 administration (Fig. 4C). In addition, the level of IL-6 in the serum of mice in the stapled + ATG-F4 group was significantly lower than that in the stapled group (Fig. 4D). Furthermore, ATG-F4 administration decreased IL-6 levels in TA and GA muscles (Fig. 4E and 4F).

### ATG-F4 Suppresses Expression of Atrophy-Related Factors (MuRF1, Atrogin-1)

**Fig. 5:**
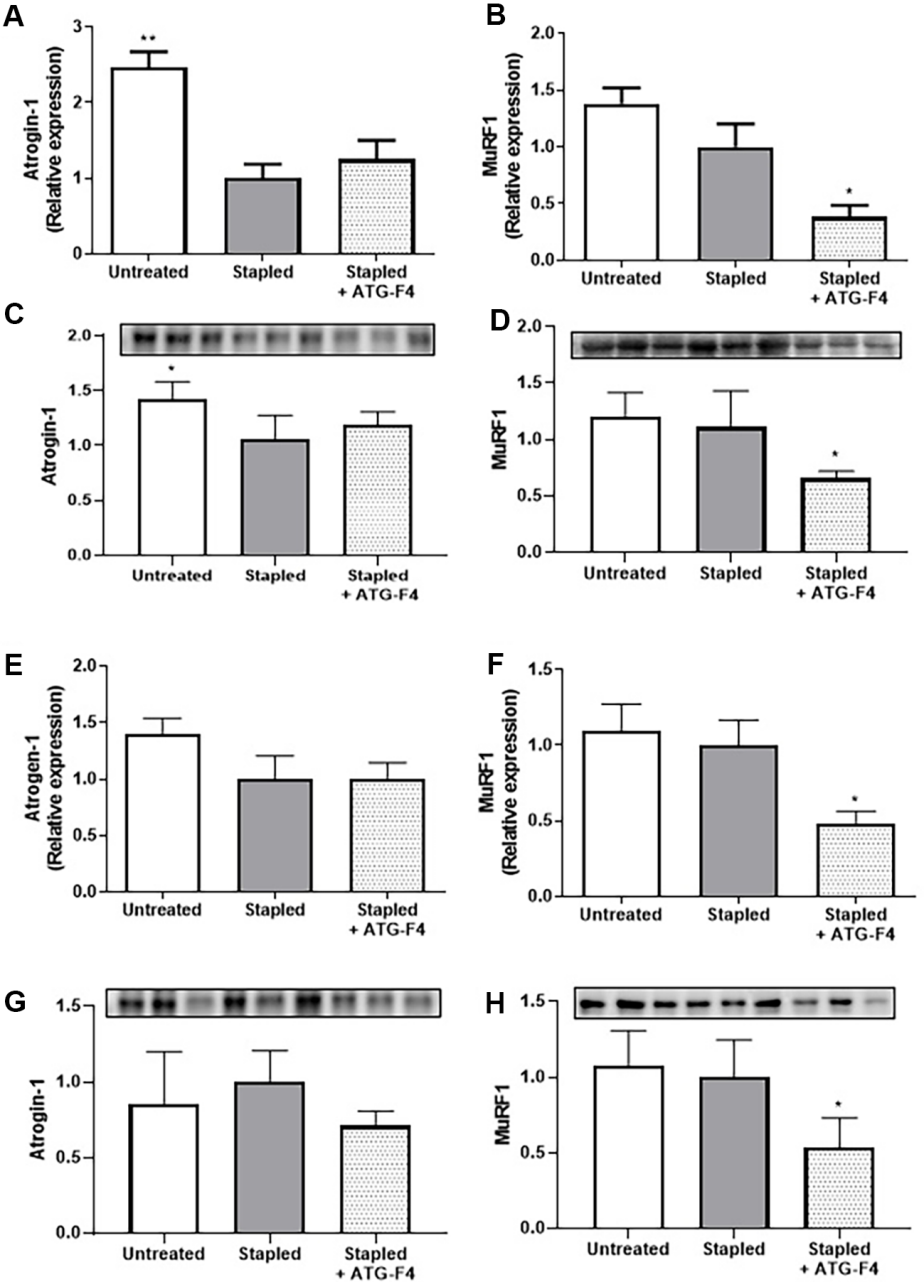
Expression of muscle atrophy factors in mice muscles. (**A–B**) mRNA and (**C–D**) protein expression of (**A** and **C**) Atrogin-1 and (**B** and **D**) MuRF1 in TA muscles; (**E–F**) mRNA and (**G–H**) protein expression of (E and G) Atrogin-1 and (F and H) MuRF1 in GA muscle. TA, tibialis anterior; GA, gastrocnemius; untreated, unstapled group; stapled, staple-induced immobilization group; stapled + ATG-F4, staple-induced immobilization + *L. reuteri* ATG-F4 (4.0×109 CFU/day)-treated group. Data are presented as the mean ± SEM (*n* = 10 mice per group). *p < 0.05 and **p < 0.01 compared to the stapled group.

The mRNA and protein expression levels of the muscle atrophy-related factors Atrogin-1 and MuRF1 in the TA and GA muscles were analyzed using RT-qPCR and western blotting, respectively. Atrogin-1 expression was significantly lower in the TA muscles of mice in the stapled group than in the untreated group. However, there was no significant difference in Atrogin-1 expression between the stapled and stapled + ATG-F4 groups (Fig. 5A and 5C). MuRF1 expression in the TA muscles of the mice in the stapled group did not differ from that in the TA muscles of the untreated group. However, MuRF1 expression was significantly lower in the TA muscles of mice in the stapled + ATG-F4 group than in the TA muscles of mice in the stapled group (Fig. 5B and 5D).

Atrogin-1 and MuRF1 expression levels did not differ significantly between the GA muscles of mice in the stapled and untreated groups (Fig. 5E and 5H). However, although Atrogin-1 expression did not differ significantly between the GA muscles of mice in the stapled + ATG-F4 and stapled groups, MuRF1 expression was significantly lower in the GA muscles of mice in the stapled + ATG-F4 group (Fig. 5F and 5H).

### ATG-F4 Activates mTOR Pathway and Protein Synthesis Signaling

**Fig. 6:**
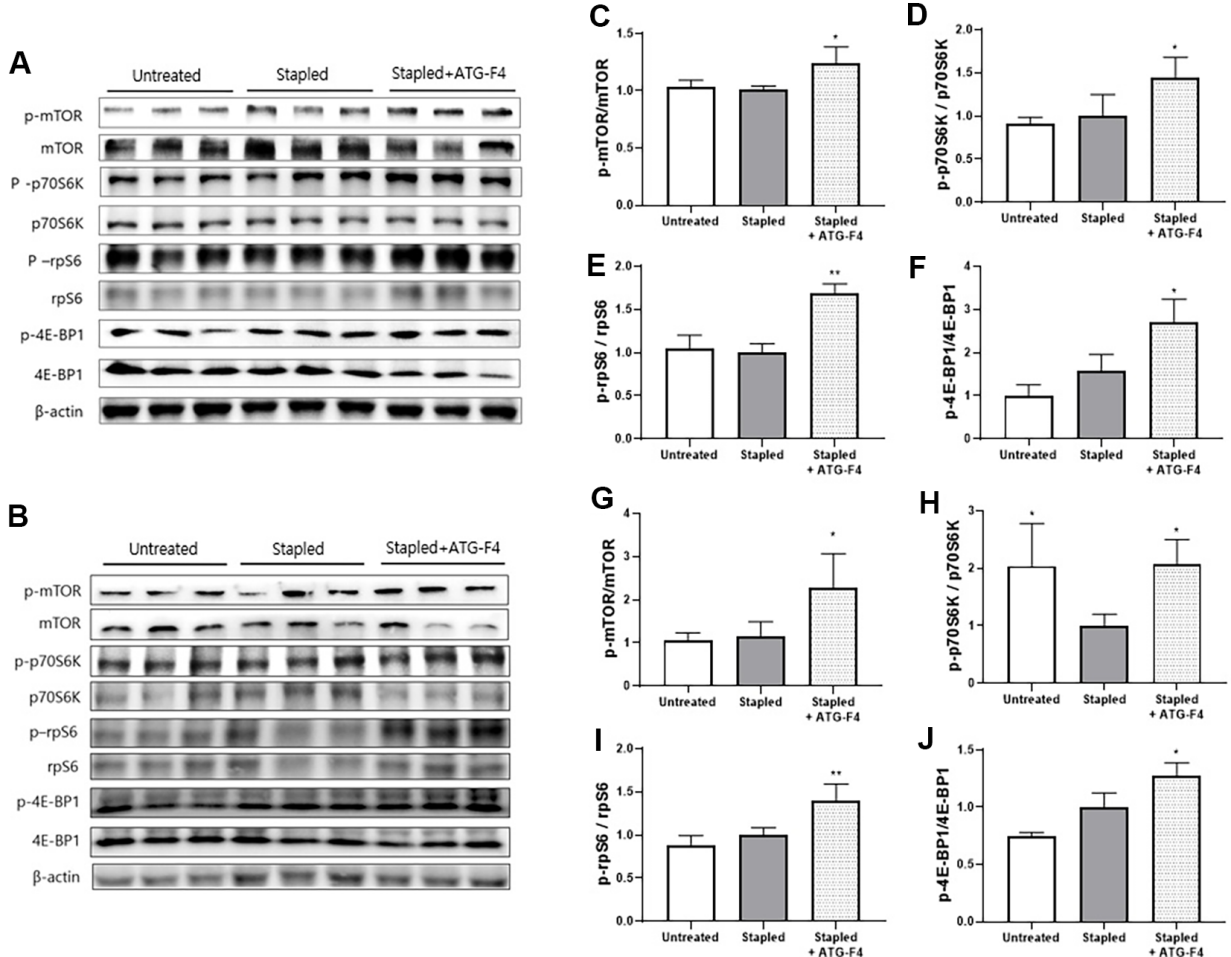
Phosphorylation of muscle synthesis proteins in mouse muscles. (**A–B**) Representative immunoblotting of (**A**) TA and (**B**) GA muscle synthesis proteins; (**C–F**) relative phosphorylation (or expression) levels of (**C**) mTOR, (**D**) p70S6K, (**E**) rpS6, and (**F**) 4E-BP1 in TA muscle; (G–J) relative phosphorylation (or expression) levels of (**G**) mTOR, (**H**) p70S6K, (**I**) rpS6, and (**J**) 4E-BP1 in GA muscle. TA, tibialis anterior; GA, gastrocnemius; untreated, unstapled group; stapled, stapleinduced immobilization group; stapled + ATG-F4, staple-induced immobilization + *L. reuteri* ATG-F4-treated group. Data are presented as the mean ± SEM (*n* = 10 mice per group). *p < 0.05 and **p < 0.01 compared to the stapled group.

We examined the effects of ATG-F4 administration on the phosphorylation of muscle synthesis-related proteins in TA and GA muscles using western blotting analysis. In the TA muscle, the phosphorylation levels of muscle synthesis-related factors, including mTOR, p70S6K, rpS6, and 4E-BP1, did not differ significantly between the stapled and untreated groups. However, ATG-F4 administration significantly increased the relative phosphorylation of these proteins in the TA muscle (Fig. 6A-6E). Similarly, in the GA muscle, muscle synthesis-related factor expression levels in the stapled group were either unchanged or decreased compared with those in the untreated group. Similar to the observations in the TA muscle, ATG-F4 administration enhanced the relative phosphorylation of muscle synthesis factors in the GA muscle (Fig. 6F-6J).

### ATG-F4 Modulates Gut Microbiota Composition

**Fig. 7:**
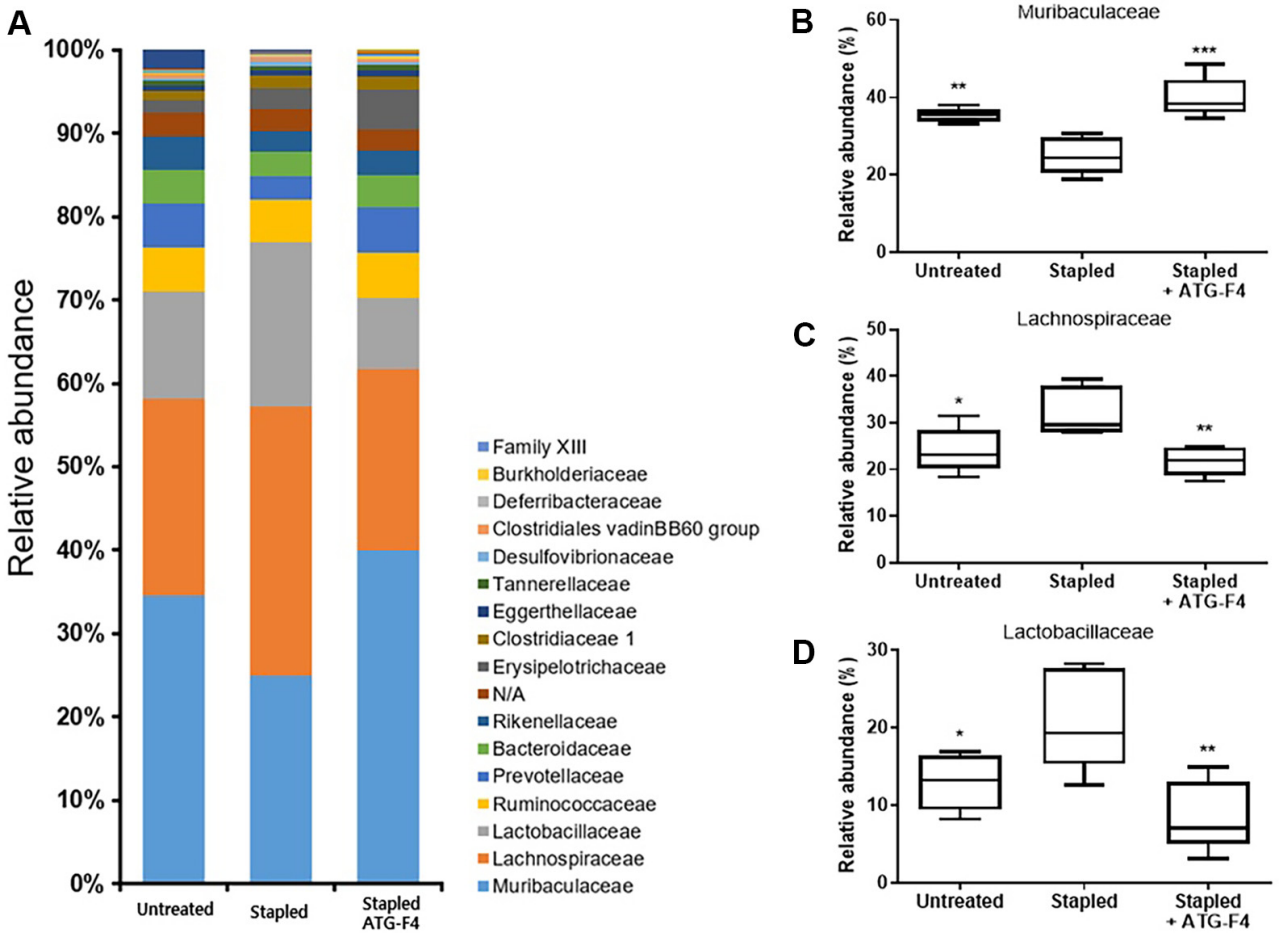
Changes in fecal bacterial community. (**A**) Stacked bar plot of the top 17 most abundant taxa (at the family level) found in fecal samples of mice from different treatments; relative abundance of (**B**) Muribaculaceae, (**C**) Lachnospiraceae, and (**D**) Lactobacillaceae. untreated, unstapled group; stapled, staple-induced immobilization group; stapled + ATG-F4, stapleinduced immobilization + *L. reuteri* ATG-F4-treated group. Data are presented as the mean ± SEM (*n* = 10 mice per group). *p < 0.05 and **p < 0.01 compared to the stapled group.

Changes in the gut microbial community were assessed by analyzing the fecal samples from each experimental group. Taxonomic abundance analysis revealed a significant increase in Bacteroidetes and a decrease in Firmicutes in the stapled + ATG-F4 group (Fig. S1). At the family level, the relative abundance of Muribaculaceae (phylum Bacteroidetes) significantly increased (Fig. 7A), whereas the relative abundance of Lachnospiraceae (phylum Firmicutes) and Lactobacillaceae (phylum Firmicutes) significantly decreased in the stapled + ATG-F4 group, similar to those in the untreated group (Fig. 7B and 7C).

### Muribaculaceae Abundance Positively Correlates with Muscle Mass

**Fig. 8:**
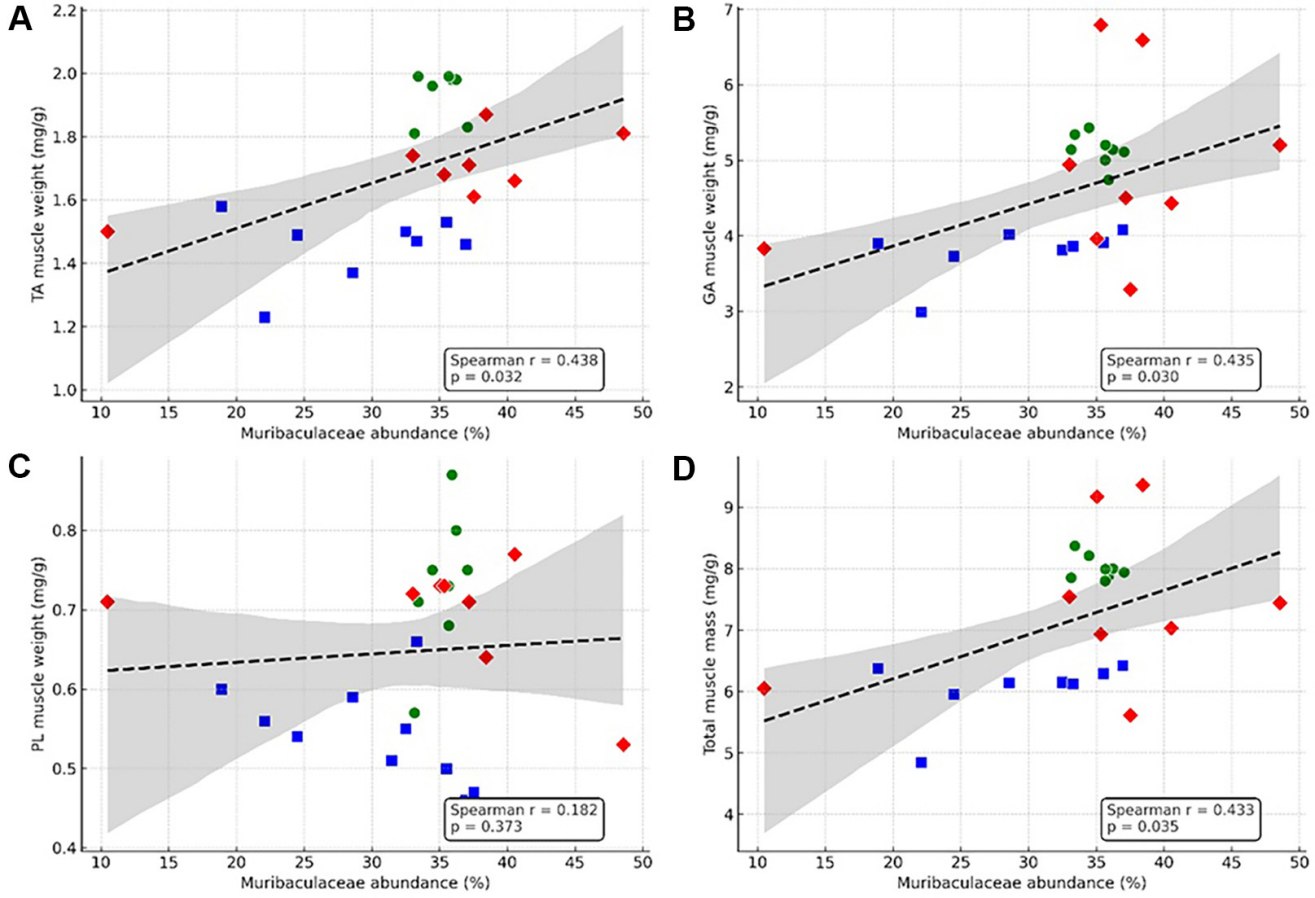
Correlation between Muribaculaceae abundance and skeletal muscle mass across different muscle groups. (**A**) TA, (**B**) GA, (**C**) PL, and (**D**) total muscle mass were analyzed for correlation with the relative abundance of Muribaculaceae in fecal microbiota. Each dot represents an individual mouse, and colors/shapes indicate experimental groups (green circles: untreated, blue squares: stapled, red diamonds: stapled + ATG-F4). Dashed black lines indicate regression lines with 95% confidence intervals (gray). *n* = 8-10 mice per group.

To investigate the relationship between the gut microbiota composition and muscle phenotype, correlations between the relative abundance of Muribaculaceae and skeletal muscle mass were assessed for each experimental group. Significant positive correlations were observed between TA, GA, and total muscle mass, with Spearman’s correlation coefficients ranging from 0.433 to 0.438 (*p* < 0.05) (Fig. 8A, 8B and 8D). However, the PL muscle mass showed no significant correlation with Muribaculaceae abundance (Spearman’s r = 0.182, *p* = 0.373) (Fig. 8C). These results suggest that the relative abundance of Muribaculaceae is associated with increased muscle mass.

### ATG-F4 Increases Circulating Butyrate and Acetate Levels

**Fig. 9:**
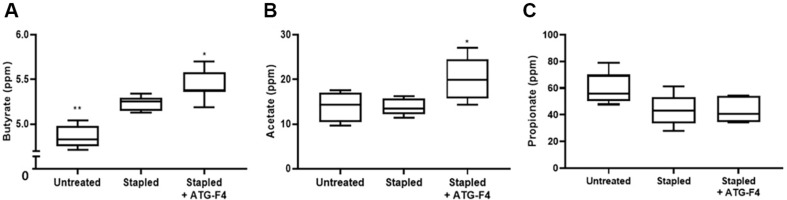
Changes in SCFA abundance in the serum of mice from different treatment groups. (**A**) Butyrate; (**B**) acetate; and (**C**) propanoate levels in mice serum. untreated, unstapled group; stapled, staple-induced immobilization group; stapled + ATG-F4, staple-induced immobilization + *L. reuteri* ATG-F4-treated group. Data are presented as the means ± SEM. *p < 0.05 and **p < 0.01 compared to the stapled group.

SCFAs, which are gut microbiome-related metabolites, were analyzed in the sera of mice using LC-MS/MS (Fig. 9). Butyrate levels were higher in mice from the stapled group than those from the untreated group, whereas mice from the stapled + ATG-F4 group showed an even greater increase in butyrate levels (Fig. 9A). In contrast, although acetate levels did not differ significantly between mice in the untreated and stapled groups, they were significantly higher in the stapled + ATG-F4 group (Fig. 9B). Propionate levels were not significantly different between the groups (Fig. 9C).

## Discussion

This study aimed to investigate the potential of *L. reuteri* ATG-F4 to ameliorate muscle atrophy and dysfunction. Our findings revealed that ATG-F4 increased the muscle fiber size, muscle mass, grip strength, and treadmill endurance in mice subjected to staple-induced immobilization.

The regulation of muscle mass involves a complex interplay between catabolic and anabolic processes. Downstream effectors of mTOR, including rpS6 kinase and eIF4E-binding protein 1 (4E-BP1), contribute to protein synthesis and muscle hypertrophy, whereas the muscle atrophy-related genes MuRF1 and Atrogin-1 mediate protein breakdown and muscle wasting [37-39]. These two pathways are influenced by the inflammatory status [40, 41]. In the present study, ATG-F4 promoted the phosphorylation of the mTOR-associated signaling pathway in the muscles and reduced MuRF1 expression. Moreover, ATG-F4 administration effectively lowered TNF-α and IL-6 levels in both blood and muscle. These results indicated that ATG-F4 enhanced muscle mass and function through anti-inflammatory and mTOR signaling pathway activation.

In contrast, we did not observe significant differences in the muscle protein levels of Atrogin-1 and MuRF1 between the untreated and stapled groups. Atrogin-1 and MuRF1 expression peaks within 12 h of treadmill exercise [42]. However, because our tissue samples were collected the day after the strength and endurance tests, it is possible that muscle atrophy markers, such as mTOR, Atrogin-1, and MuRF1, may have already returned to baseline. Nevertheless, ATG-F4 significantly enhanced the phosphorylation of protein synthesis-related factors, specifically mTOR, p70S6K, and rpS6, and reduced MuRF1 expression. These findings suggest that ATG-F4 effectively promotes anabolic signaling and suppresses catabolic activity even during muscle recovery. Future experiments are required to investigate the effect of the timing of tissue collection on the analysis of these markers.

Muscle-related diseases, including myasthenia gravis, spinal cord injury, cancer cachexia, and sarcopenia, markedly influence microbial communities in the gut [43-46]. Although the effects of immobilization to induce physical muscle atrophy on gut microbiota composition have not been thoroughly investigated, mice with staple-induced immobilization exhibited a microbial profile different from that of untreated control mice in this study. Specifically, the observed elevation of TNF-α and IL-6 levels in both serum and muscle tissues of the stapled group indicated the presence of local inflammation.

Notably, the gut microbiota composition, which was altered by ATG-F4 administration in the stapled group, resembled that observed in healthy animals. ATG-F4 administration led to an increase in Muribaculaceae and a decrease in Lachnospiraceae and Lactobacillaceae, resulting in a microbial composition similar to that of the untreated group. Muribaculaceae abundance is generally reduced in inflammation-related diseases such as antibiotic-induced colitis, postoperative neuroinflammation, and inflammatory bowel disease [47-49], whereas Lachnospiraceae and Lactobacillaceae either increase or decrease depending on the specific inflammation model [50-52].

To explore the link between changes in gut microbiota and muscle preservation, we calculated the correlation between the relative abundance of Muribaculaceae and muscle mass across various muscle groups. The abundance of Muribaculaceae was positively correlated with the mass of the TA and GA muscles, as well as the total muscle mass. This suggests that Muribaculaceae enrichment may contribute to muscle maintenance.

Muribaculaceae, Lachnospiraceae, and Lactobacillaceae were the dominant bacterial families in all three groups, collectively comprising approximately 70% of the total gut microbiota [53]. These bacteria can ferment complex carbohydrates and dietary fibers, producing SCFAs as metabolic byproducts [54]. SCFAs play a crucial role in maintaining gut function by serving as energy sources for colonocytes, reducing inflammation, and enhancing intestinal motility [55-57]. SCFAs also exert beneficial effects on skeletal muscle metabolism and function [58, 59].

In this study, the serum levels of SCFAs, such as butyrate and acetate, in the ATG-F4 administered mouse group were significantly elevated, with an increase in Muribaculaceae, a known SCFAs producer. Concomitantly, TNF-α and IL-6 levels in both serum and muscle tissue in the staple-induced immobilization mice were reduced by ATG-F4 administration. Many studies have reported the beneficial effects of gut microbiota-induced butyrate. Walsh *et al*. reported the beneficial effect of butyrate on muscle mass by inhibiting histone deacetylase (HDAC), which is one of the targets of muscle atrophy treatment [60]. In a study using old mice with pre-sarcopenic senescent accelerated mouse prone 8, daily administration of a SCFA cocktail (acetate, butyrate, and propionate) attenuated fat infiltration and improved mitochondrial biogenesis of atrophic muscle, and activated the AKT/mTOR/S6K1 and AMPK/PGC1α pathways was observed [61, 62]. Zhao *et al*. reported that SCFA, such as sodium butyrate, prevented muscle atrophy by regulating inflammatory reactions and activating signaling pathways in dexamethasone-induced skeletal muscle atrophy. Isogai *et al*. have reported that butyrate protected against renal crystal formation by reducing inflammation and microvillar damage [63]. Acetate also supports muscle function by promoting glycogen replenishment and increasing acetyl-CoA concentrations in the skeletal muscles [64, 65].

Although we could not confirm that the SCFAs resulting from gut microbiota changes directly enhanced muscle function, we speculate that the preventive effects of ATG-F4 resulted from the mitigation of inflammation by modulating the gut microbiota and increasing SCFAs levels.

## Conclusion

In this study, ATG-F4 significantly enhanced muscle strength and exercise performance in an immobilized mouse model of muscle atrophy by increasing muscle mass and fiber size. Its primary mechanism appears to involve the suppression of systemic inflammation, which is a key contributor to muscle degradation. This effect is likely mediated by the modulation of the gut microbiota, particularly through the enrichment of beneficial SCFA-producing bacteria. SCFAs exert anti-inflammatory effects and protect against muscle atrophy. This pathway activates mTOR signaling, enhances protein synthesis, and reduces the expression of atrophy-related factors. Although ATG-F4 shows promise as a prophylactic or therapeutic agent, further research is required to clarify the causal links among inflammation, microbiota modulation, and muscle preservation.

## Supplemental Materials

Supplementary data for this paper are available on-line only at http://jmb.or.kr.
