# Supplementary material for: Limosilactobacillus reuteri ATG-F4 Improves the Muscle Strength and Muscle Mass of Mice with Immobilization-Induced Muscular Atrophy
Source: J Microbiol Biotechnol. 2025 Oct 15;35:e2506004. doi: 10.4014/jmb.2506.06004 (PMC12549228; doi:10.4014/jmb.2506.06004)
Supplement: Supplementary file 1 [file jmb-35-e2506004-supple.pdf]

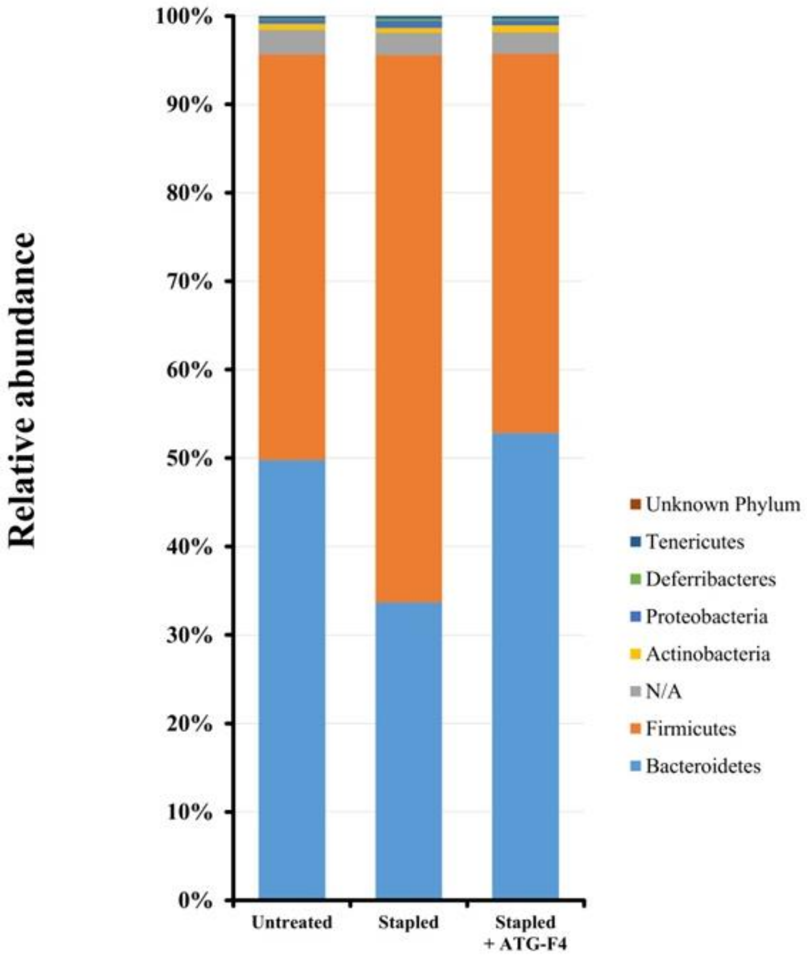

Supplementary Fig. 1. Relative abundance of bacterial taxa at the phylum level in fecal samples from each experimental group.
